# Resting-State Pallidal-Cortical Oscillatory Couplings in Patients With Predominant Phasic and Tonic Dystonia

**DOI:** 10.3389/fneur.2018.00375

**Published:** 2018-05-31

**Authors:** Fusako Yokochi, Kenji Kato, Hirokazu Iwamuro, Tsutomu Kamiyama, Katsuo Kimura, Akihiro Yugeta, Ryoichi Okiyama, Makoto Taniguchi, Satoko Kumada, Junichi Ushiba

**Affiliations:** ^1^Department of Neurology, Tokyo Metropolitan Neurological Hospital, Tokyo, Japan; ^2^Department of Biosciences and Informatics, Faculty of Science and Technology, Keio University, Kanagawa, Japan; ^3^Department of Neurosurgery, Tokyo Metropolitan Neurological Hospital, Tokyo, Japan; ^4^Department of Pediatric Neurology, Tokyo Metropolitan Neurological Hospital, Tokyo, Japan

**Keywords:** dystonia, globus pallidus, motor cortex, local field potentials, coherence, resting-state, deep brain stimulation

## Abstract

Pallidal deep brain stimulation (DBS) improves the symptoms of dystonia. The improvement processes of dystonic movements (phasic symptoms) and tonic symptoms differ. Phasic symptoms improve rapidly after starting DBS treatment, but tonic symptoms improve gradually. This difference implies distinct neuronal mechanisms for phasic and tonic symptoms in the underlying cortico-basal ganglia neuronal network. Phasic symptoms are related to the pallido–thalamo–cortical pathway. The pathway related to tonic symptoms has been assumed to be different from that for phasic symptoms. In the present study, local field potentials of the globus pallidus internus (GPi) and globus pallidus externus (GPe) and electroencephalograms from the motor cortex (MCx) were recorded in 19 dystonia patients to analyze the differences between the two types of symptoms. The 19 patients were divided into two groups, 10 with predominant phasic symptoms (phasic patients) and 9 with predominant tonic symptoms (tonic patients). To investigate the distinct features of oscillations and functional couplings across the GPi, GPe, and MCx by clinical phenotype, power and coherence were calculated over the delta (2–4 Hz), theta (5–7 Hz), alpha (8–13 Hz), and beta (14–35 Hz) frequencies. In phasic patients, the alpha spectral peaks emerged in the GPi oscillatory activities, and alpha GPi coherence with the GPe and MCx was higher than in tonic patients. On the other hand, delta GPi oscillatory activities were prominent, and delta GPi–GPe coherence was significantly higher in tonic than in phasic patients. However, there was no significant delta coherence between the GPi/GPe and MCx in tonic patients. These results suggest that different pathophysiological cortico-pallidal oscillations are related to tonic and phasic symptoms.

## Introduction

Dystonia is defined as a movement disorder characterized by sustained or intermittent muscle contractions causing abnormal, often repetitive, movements, postures, or both (1). Dystonia is known to be characterized by tonic symptoms of abnormal sustained co-contractions between agonist and antagonist muscles, often accompanied by regular or irregular phasic symptoms, such as tremor, myoclonus, or twisted involuntary movements (2–5). Globus pallidus internus (GPi)-deep brain stimulation (DBS) is an established and highly effective neurotherapeutic intervention for severe dystonia (6–12). It is commonly accepted that predominant phasic symptoms reduce rapidly after GPi-DBS intervention, whereas predominant tonic symptoms improve gradually over a year (13, 14). From these clinical observations, it can be speculated that differential neuronal mechanisms may underlie each of the clinical phenotypes (i.e., predominant phasic or tonic symptoms). A few articles have reported that the phasic symptoms are related to the low frequency band (4–10 Hz) and the pallido–thalamo–cortical pathway. However, it is not yet clear that marginal frequency band is related to tonic symptoms, and whether the pallido–thalamo–cortical pathway participates. The basal ganglia (BG) do have an important influence on the motor cortex, but how a BG abnormality could produce dystonia is not completely clear. In the classic view, there are two anatomical pathways through the BG. The direct pathway (striatum-internal division of the globus pallidus-thalamus) is facilitatory and the indirect pathway (striatum-external division of the globus pallidus subthalamic nucleus-internal division of the globus pallidus-thalamus) is inhibitory. The direct pathway helps directly the desired movement, while the indirect pathway inhibits unwanted movements. It is supposed that there is an imbalance in the direct and indirect pathways so that the direct pathway is relatively overactive (or that the indirect pathway is relatively underactive) (15). The postulated imbalance should lead to excessive movement and, in particular, loss of surround inhibition. Is this circuit related to the tonic symptoms in dystonia?

Using the opportunities provided by DBS postoperative recording of local field potentials (LFPs) in the GPi, the increasing body of evidence suggests that excessive pallidal oscillatory activity over the low frequency band (4–10 Hz) is predominant in patients with primary dystonia (16, 17). Excessive pallidal low-frequency oscillation is positively correlated with the level of involuntary dystonic muscle activity (18). More importantly, the previous research found that the low-frequency coherence only existed between the GPi LFPs and the rhythmic involuntary muscle activity observed in phasic dystonic patients, but not the sustained hypertonic activity observed in tonic dystonic patients, suggesting that GP oscillations differentially reflect each clinical phenotype of dystonia (4). In addition, it has been found that the pallidal low-frequency oscillatory activity is suppressed during GPi high-frequency stimulation, especially in patients with predominant phasic dystonic movements, supporting a causal link between improvement in phasic symptoms and reduction of low-frequency pallidal activity (5).

One of the most challenging problems in the field of the pathophysiology of dystonia is the heterogeneous nature of the dystonic movements. It has become common to investigate the abnormal brain networks in patients with movement disorders using LFP electrophysiological techniques in Parkinson’s disease (PD) (19–21). Recently, the use of functional magnetic resonance imaging techniques in the investigation of task-specific focal dystonia has been re-evaluated (22), since it provides the possibility to investigate neural networks without contamination by involuntary dystonic movements.

Since the patients need not perform any voluntary movements in the resting state, resting-state functional connectivity may show the inherent neural networks that are not confounded by differences in complex involuntary dystonic movements. Using this technique, imaging studies have suggested that abnormal increased activation in the sensorimotor cortex and BG is related to the reduction in resting-state connectivity within both the sensorimotor and BG networks in task-specific focal hand dystonia (23).

Thus, investigating resting-state cortico-pallidal networks may elucidate the inherent properties of the clinical phenotypes, such as the predominant phasic or tonic symptoms. To identify the distinct symptom-related characteristics in resting-state pallidal-motor cortex (MCx) connectivity, LFPs implanted from DBS electrodes in the bilateral GPi and globus pallidus externus (GPe) and electroencephalograms (EEGs) over the bilateral MCx were simultaneously recorded in 19 dystonic patients. Of the 19 dystonic patients, 10 were diagnosed as having predominant phasic symptoms (phasic patients), while the other 9 patients were diagnosed as having predominant tonic symptoms (tonic patients). Then, the resting-state GPi and GPe oscillatory activities were analyzed over the delta (2–4 Hz), theta (5–7 Hz), alpha (8–13 Hz), and beta (14–35 Hz) frequencies in each clinical subtype of dystonia. Furthermore, the resting-state functional couplings between the GPi, GPe, and MCx were calculated using coherence analysis to investigate the symptom-related differences in functional connectivity across the GPi, GPe, and MCx. The results showed that the phasic symptoms were related to the alpha spectral peaks that emerged in the GPi oscillatory activities, and the alpha GPi coherence with the GPe and MCx was clear. On the other hand, the delta GPi oscillatory activities were prominent in the tonic symptoms, with no significant delta coherence between the GPi/GPe and MCx. These results suggest different pathophysiological cortico-pallidal oscillations related to the tonic and phasic symptoms. The tonic symptoms were correlated to the delta oscillatory activities, and the phasic and tonic symptoms have different circuits.

## Patients and Methods

### Patients

The present study was approved by the ethics committees of the Tokyo Metropolitan Neurological Hospital (Tokyo, Japan) and the Faculty of Science and Technology, Keio University (Kanagawa, Japan). The study and publication were explained to all patients, and written, informed consent was obtained from all patients.

Nineteen patients with dystonia treated by bilateral GPi DBS were included in this study (10 male, 9 female; mean age 42.3 ± 16.4 years; mean disease duration 9.9 ± 8.6 years), as shown in Table 1; 17 had primary dystonia, and 2 had tardive dystonia, with 14 having generalized dystonia, 4 having focal dystonia, and 1 having segmental dystonia. All of nine patients classified into the tonic patients had severe truncal dystonia. In 10 patients classified into the phasic patients, the main symptoms were phasic dystonic movements. The five patients in the phasic patients had slight tonic symptoms of trunk. The tonic symptoms of the shoulder girdle or neck were observed in three phasic patients. Two phasic patients had facial phasic movements with slight tonic symptoms.

**Table 1:** Clinical details of patients with predominant phasic and tonic symptoms of dystonia.

	Patient	Sex	Age (years)	Disease duration (years)	Clinical diagnosis/distribution	BFMDRS score before surgery	Tonic symptoms total (r/m)	Phasic symptoms total (r/m)
Phasic group	1	F	43	36	C, F/DYT11	10	2	6
	2	F	63	2	C, F/tardive	17	3	6
	3	F	44	17	C, G	27	3	7
	4	M	35	7	C, S	27	3	7
	5	M	66	9	C, G	23	2	7
	6	F	56	3	C, G	17	3	8
	7	M	71	6	C, F/tardive	18	3	7
	8	M	53	23	C, F	8	3	7
	9	M	44	13	C, G	12	3	7
	10	F	49	9	C, G	15	3	6

Average (SD)			52.4 (11.5)	12.5 (10.4)		17.4 (6.6)	2.8 (0.4)	6.8 (0.6)

Tonic group	11	F	23	2	I, G	66	7	2
	12	M	32	14	I, G	15	7	0
	13	M	28	2	I, G	61	6	0
	14	F	12	8	C, G	62	7	2
	15	M	38	3	C, G	44	7	0
	16	F	25	16	I, G/DYT1	50	8	1
	17	M	55	7	C, G	18	7	4
	18	M	47	4	I, G	63	7	0
	19	F	19	8	I, G	28	7	3

Average (SD)			31 (13.7)	7.1 (5.1)		45.2 (20.2)	7.0 (0.5)	1.3 (1.5)

*I, isolated dystonia; C, combined dystonia; F, focal dystonia; G, generalized dystonia; S, segmental dystonia; tardive, tardive dystonia; BFMDRS, Burke-Fahn-Marsden Dystonia Rating Scale; Total (r/m), total scores during resting and movement*.

Phasic and tonic symptoms were observed in most patients, but the degrees of the two clinical expressions were different in each patient. Overall clinical symptoms of dystonia were evaluated using the Burke-Fahn-Marsden Dystonia Rating Scale (BFMDRS) by two neurologists (Fusako Yokochi and Katsuo Kimura) who are specialists in movement disorders and stereotactic surgical treatment. Furthermore, to divide the patients into the two groups depending on the two specific symptoms, sustained muscle contractions (tonic symptoms) and intermittent muscle contractions (phasic symptoms), the symptoms of each patient were evaluated by the following methods. Since no general scoring has been developed to evaluate phasic and tonic symptoms, a scoring method was developed, as follows. The two clinical symptoms were observed in the resting state in the supine or sitting position and while performing simple movements (elevation of bilateral upper limbs, flexion of the right or left knee joint, or opening and closing of the mouth or eyes). Not only visual inspection but also surface EMGs were recorded from the bilateral orbicularis oris, sternocleidomastoid, deltoid, thoracic paravertebral, biceps brachii, triceps brachii, and tibialis anterior muscles to check the discharges of intermittent muscle contractions. Twitch muscle contractions were excluded from the evaluation. Tonic and phasic symptoms were separately evaluated with five levels linked to commonly accepted clinical terms: 0 = normal, 1 = slight, 2 = mild, 3 = moderate, and 4 = severe. When the total values of the scores of tonic or phasic symptoms in the two conditions were greater than five points, tonic or phasic symptoms, respectively, were classified as dominant.

In 10 patients, the average total phasic scores (6.8 ± 0.6) were higher than the average total tonic scores (2.8 ± 0.4), and in 9 patients, the average total tonic scores (7.0 ± 0.5) were higher than the average total phasic scores (1.3 ± 1.5), as shown in Table 1. The patient group with the high phasic scores was the phasic group, and who with the high tonic scores was the tonic group. Age and disease duration were 52.4 ± 11.5 and 12.5 ± 10.4 years, respectively, in the phasic group, and 31.0 ± 13.7 and 7.1 ± 5.1 years, respectively, in the tonic group. The two groups were sex-matched, although the patients in the tonic group were significantly younger than those in the phasic group (*p* < 0.005). The BFMDRS scores were significantly higher in the tonic group than in the phasic group (*p* < 0.001). The phasic group included patients with focal type and generalized type. LFP profiles in the focal and generalized types in the phasic group were similar (Figure S3 in Supplementary Material). Therefore, LFPs of focal and generalized dystonia with severe phasic symptoms were summarized as the phasic group and analyzed. In addition, there was no significant difference in LFP profiles between sexes (*p* = 0.233, unpaired *t*-test). All patients took drugs such as trihexyphenidyl, clonazepam, or others. All drugs were stopped from the day before the operation until after the LFP examination. No patients had botulinum toxin therapy in the 1 year before the operation.

### Surgical Procedures

Deep brain stimulation electrodes were implanted bilaterally into the GP under general anesthesia in all patients. Stereotactic surgery was performed using a Leksell stereotactic frame (Elekta Instruments, Norcross, GA, USA) and a microelectrode recording system (Medtronic Neurological Division, Minneapolis, MN, USA). The target coordinates were determined from T2-weighted magnetic resonance imaging (MRI) as 2.0 mm anterior to the midpoint of the anterior commissure–posterior commissure (ACPC) line (*Y*) and 5.0 mm inferior to the ACPC line (*Z*). The laterality coordinate (*X*) was 20 ± 1.3 mm lateral from the midline and corresponded to the position of the optic tract (OT) identified on the coronal section image of MRI fast spin echo/inversion recovery. The track was selected along the GP on the coronal section by visual identification, passing the dorsal surface of the OT. Microelectrode recordings were performed one track at a time and started 30 mm superior to the target point. The putamen, GPe, and GPi were distinguished by laminas: the lamina medullaris lateralis between the putamen and GPe and the lamina medullaris medialis between the GPe and GPi. The neuronal activities in the lamina were less or absent compared to activities in the GPe and GPi. After detection of the OT, the distance between the activities of the OT and the globus pallidus internus was defined by the response to a flashlight. The electrical stimuli (5.0 mA, 60 µs, 130 Hz) were applied through the recording electrode, and the presence of abnormal tonic contractions was observed. The appropriate track was selected along each length of GPi and GPe neuronal activities for about 5 mm along the track without side effects. The DBS electrodes (Model 3387 or 3389; Medtronic), which have four contacts, were implanted therein. Contact 0, the most ventral contact, was placed in the GPi, and contact 3, the most dorsal contact, was placed in the GPe. The ventral edge of the lowest contact 0 was put 2 mm from the OT. The positions of the four contacts were contacts 0 and 1 in the GPi, and contacts 2 and 3 in the GPe. Contact locations were confirmed on postoperative MRI (Figure S1 in Supplementary Material).

The mean lengths of the left and right GPes were 6.5 ± 1.1 ± and 6.0 ± 1.4 mm, and those of the left and right GPis were 5.1 ± 2.1 and 5.0 ± 1.7 mm. The mean angles of the right and left tracks were 4.3 ± 3.6 degrees to the midline and 63.9 ± 3.9 degrees to the ACPC line, respectively (Table S1 in Supplementary Material).

### Postoperative LFPs, EEG, and EMG Recording

Two days after the operation and before the implantation of pulse generators, LFPs were recorded from the four contacts of each DBS electrode referenced to linked ears. EEGs were recorded from the bilateral sensorimotor areas (C3/C4 and FC3/FC4) according to the 10/20 system using Ag/AgCl disk electrodes, referenced to the ears. The impedance of all channels was kept below 10 kΩ throughout recording. In two patients (patients 8 and 15), EEG recordings could not be performed due to surgical dressings. LFPs and EEG data were band-pass filtered over 1–150 Hz and digitized (sampling frequency 1,000 Hz) using a biosignal recorder (Neurofax EEG 1200; Nihon Kohden Corporation, Tokyo, Japan). Bipolar derivation of adjacent DBS contact pairs 0–1, 1–2, and 2–3 was calculated for offline analysis [Mathworks, MA, USA]. As for EEG recording, bipolar derivation of FC3-C3 and FC4-C4 was performed for the offline analysis to minimize volume conduction. Surface EMGs were also recorded using pairs of Ag/AgCl disk electrodes attached with an interelectrode distance of 20 mm to the muscle bellies of the following muscles: sternocleidomastoid, biceps brachii, triceps brachii, flexor carpi radialis, and extensor digitorum communis. Each muscle was recorded bilaterally. Involuntary movements such as to inhibit recording were not observed during the examination. Patients were instructed to lie in the supine position and to be awake in the relaxed condition with eyes closed for around 10 min. During the resting-state recordings, EMG recordings were carefully monitored to check whether they showed involuntary or voluntary movements. This awaking state without involuntary and voluntary movements was defined as the resting-state. Finally, the LFP and EEG data over 5 min in which any involuntary or voluntary EMG activities were not included were analyzed.

### Power Spectra

To investigate the resting-state power spectra of GP LFPs, the discrete Fourier transform was used for the power spectral analysis, based on the method of Halliday et al. (24). Power spectra were calculated by dividing the waveform signal into 300 sections of the same duration of 1,024 ms (1,024 data points without overlap), yielding a frequency-resolution of 0.987 Hz. Each section was Hanning-windowed to minimize spectral leakage. LFPs were calculated for the three bipolar contact pairs of the DBS electrodes. After estimating the GPi and GPe power spectra, the spectral peaks were calculated over the frequency range of 2–35 Hz, based on the method established by Solages et al. (25) with modification by Kato et al. (20). In brief, spectral peaks in the theta, alpha, and beta frequency ranges were defined as follows: (1) each spectral peak had to be higher than the mean power values of the six bins below and the six bins above and (2) for detecting a peak at low frequencies of 6, 5, and 4 Hz, the number of bins for the calculation corresponded to five, four, and three bins, respectively, instead of six bins. The power spectra of 2–35 Hz were averaged on sub-frequency bands defined as 2–4 Hz (delta), 5–7 Hz (theta), 8–13 Hz (alpha), and 14–35 Hz (beta) according to the previous studies (delta (26), theta, alpha, and beta (27)). The significance of the power GPi and GPe spectral difference in each frequency in all the patients was tested using two-factor analysis of variance (ANOVA) (i.e., GPi/GPe × frequency bands). The differences in the resting power spectra between the two phasic and tonic symptom groups and between GPi and GPe oscillatory activities in each frequency band were tested using three-factor ANOVA (i.e., GPi/GPe × frequency bands × phasic/tonic). Finally, Pearson correlation coefficients were computed between the values of spectral power in the GPi and GPe over each frequency range (delta, theta, alpha, and beta) and the BFMDRS scores across patients. The significance level was Bonferroni-corrected for multiple comparisons.

### Coherence

To investigate the functional couplings over the delta, theta, alpha, and beta frequency ranges, coherence was evaluated between the following couplings: (1) GPe and ipsilateral GPi (GPi–GPe); (2) GPe and ipsilateral MCx (GPe–MCx); and (3) GPi and ipsilateral MCx (GPi–MCx). Coherence (24, 28) was used to estimate the linear correlation between simultaneously recorded data, in which a value of 1 indicates perfect linear correlation. Correlation in each coupling was calculated by coherence using the following equation (20, 27)

(1) $$C_{xy}^{2}(f)=\frac{{\left|\bar{P_{xy}(f)}\right|}^{2}}{\bar{P_{xx}(f)}\cdot \bar{P_{yy}(f)}},$$

where $\bar{P_{xx}(f)}$ and $\bar{P_{yy}(f)}$ are the averaged autospectra of the signals *X* and *Y* throughout the segments for a given frequency *f*, respectively. $\bar{P_{xy}(f)}$ is the average cross-spectrum between these two parameters throughout the segments. To clarify the significance level, coherence values were also calculated using the shuffled time-series data as described by Kato et al. (20, 27). Briefly, 10,000 pairs of surrogate data that were randomly permutated for every 1,024 data points were created. Then, the mean coherence values across the 10,000 pairs were set as the significance level in each coupling. The differences in the resting-state GPi–MCx, GPe–MCx, and GPi–GPe coherence values between the two phasic and tonic symptom groups and over frequency bands were tested using three-factor ANOVA. Finally, Pearson correlation coefficients were computed between values of GPi–MCx, GPe–MCx, and GPi–GPe coherence over each frequency range (i.e., delta, theta, alpha, and beta) and the BFMDRS scores across patients. The significance level was Bonferroni-corrected for multiple comparisons.

## Results

During this examination, patients were in the resting sate and had no severe tonic and phasic symptoms on visual observation and on EMG recordings, which is the micro lesion effect (MLE). Improvement of clinical symptoms in patients with PD and dystonia treated by DBS was observed before starting DBS stimulation, and this phenomenon, called MLE, is reported in patients with dystonia (29). The BFMDRS scores with implanted electrodes for 3 weeks were significantly decreased by 30% in comparison with the scores before the operation.

The summarized results are as follows. The phasic symptoms related to the alpha spectral peaks emerged in the GPi oscillatory activities, and alpha GPi coherence with both the GPe and MCx was significant. On the other hand, the delta GPi oscillatory activities were prominent in the tonic symptom group, with no significant delta coherence between the GPi/GPe and MCx. The regression analysis showed that there were no significant correlations between GPi/GPe/MCx power spectral or their functional couplings and BFMDRS scores in all the patients in each frequency band (*p* = 0.155–0.894). These results suggest different pathophysiological cortico-pallidal oscillations related to the tonic and phasic symptoms. These results show that the tonic symptoms are correlated to the delta oscillatory activities, and the phasic and tonic symptoms have different circuits.

### Oscillatory Activities in Internal and External Segments of the Globus Pallidus

**Figure 1:**
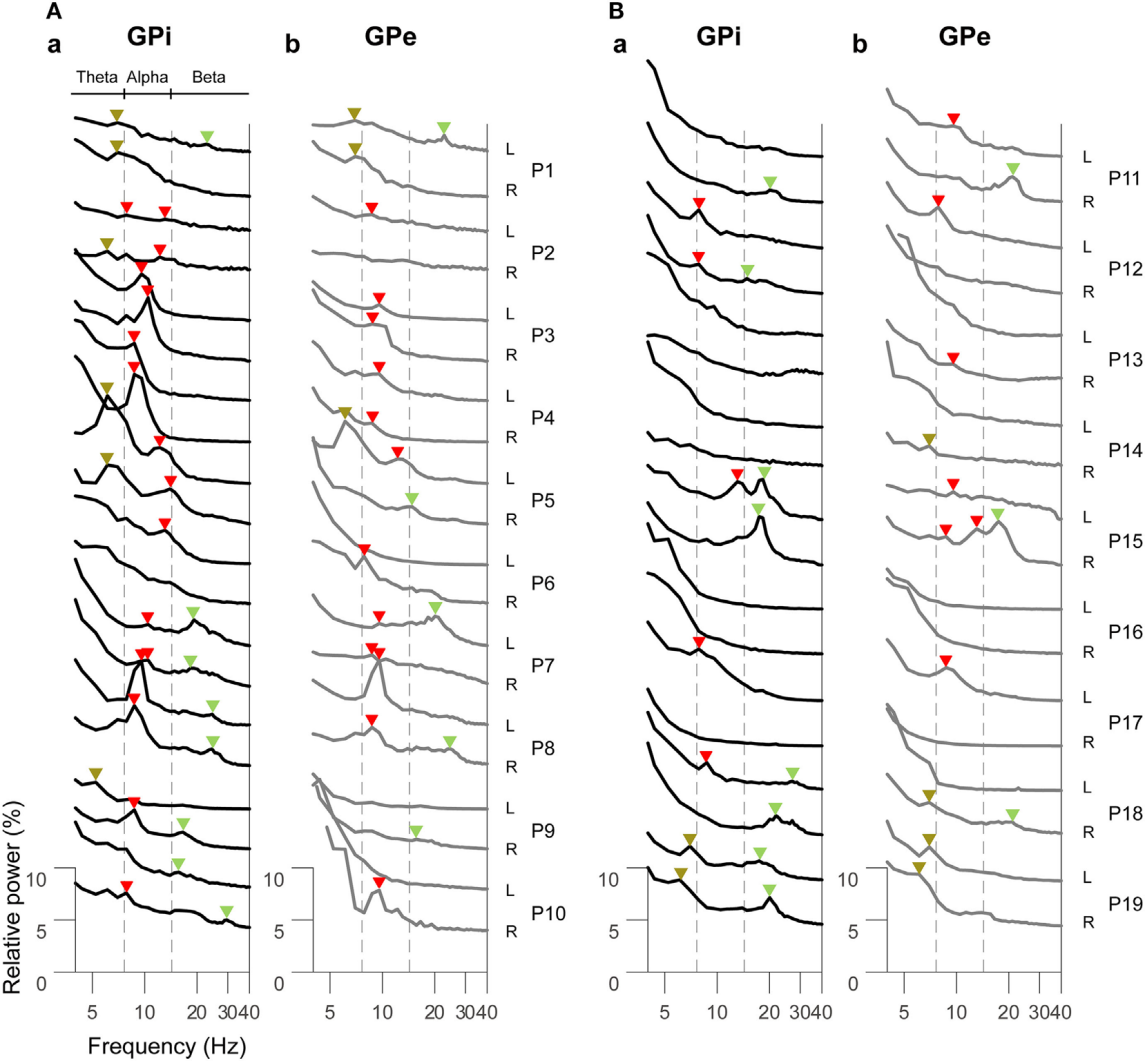
Resting-state power spectral profiles in the bilateral GPi and GPe in phasic **(A)** and tonic **(B)** patients. The black and gray solid lines represent the GPi (a) and GPe (b) power spectra over the theta (5–7 Hz), alpha (8–13 Hz), and beta (14–35 Hz) frequency bands, respectively. Patient numbers and the recorded hemispheres (L: left hemisphere and R: right hemisphere) are shown in the right of each power spectral profile. The brown, red, and green arrows shown above each power spectral profile represent the spectral peaks over the theta, alpha, and beta bands, respectively. There is no spectral peak in the delta (2–4 Hz) band. The vertical dotted gray lines show the boundaries of each frequency range (between theta and alpha, and between alpha and beta bands).

The resting-state oscillatory activities in the GPi and GPe over 2–40 Hz across all dystonic patients (38 hemispheres) showed that the GPi power was more prominent than GPe power over the alpha frequency (8–13 Hz) band (*p* < 0.01, Figure S2 in Supplementary Material), suggesting that frequency-specific distinct oscillatory patterns between the GPi and GPe are present at rest. Next, the GPi/GPe power spectral profiles were divided into the clinical phenotypes of predominant phasic (Figure 1A) or tonic (Figure 1B) dystonic symptoms. Figure 1 shows that the prominent spectral peaks over the alpha frequency range are observed more in the phasic patients (i.e., 16/12 peaks in the GPi/GPe) than in the tonic patients (i.e., 5/7 hemispheres in the GPi/GPe). Within the phasic patients, the alpha spectral peaks were more observed in the GPi than in the GPe. As for the other frequency ranges, there was no clear difference between predominant phasic [delta (2–4 Hz) 0/0, theta (5–7 Hz) 6/3, and beta (14–35 Hz) 8/5 peaks in the GPi/GPe, respectively] and tonic (i.e., delta 0/0, theta 2/5, and beta 8/3 peaks in the GPi/GPe, respectively) patients. These results suggest that the resting-state alpha GPi/GPe oscillatory activities are characteristic to phasic patients.

**Figure 2:**
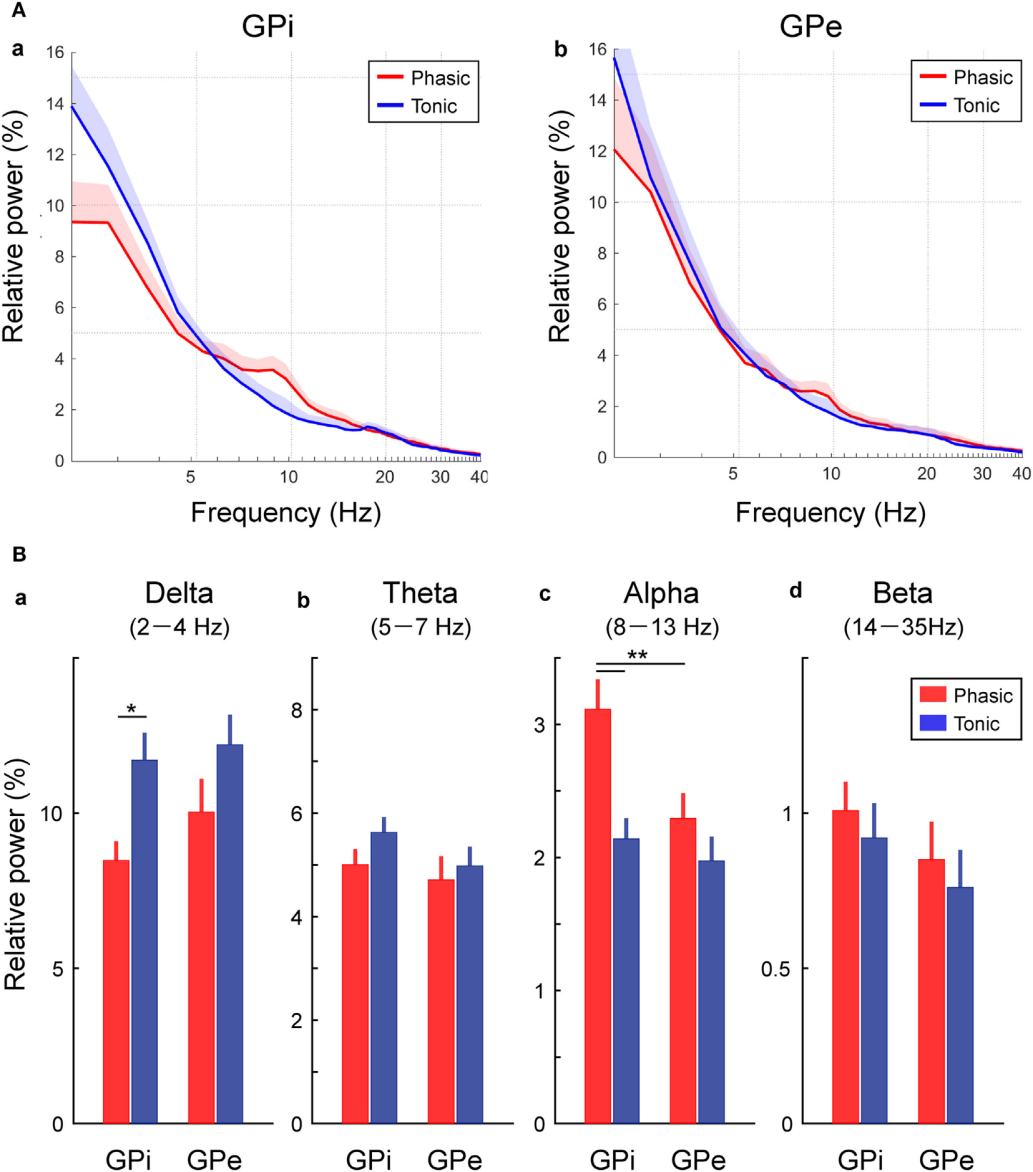
Frequency-specific GPi and GPe oscillatory activities in phasic and tonic patients. **(A)** The averaged relative power in the GPi (a) and GPe (b) over the delta (2–4 Hz), theta (5–7 Hz), alpha (8–13 Hz), and beta (14–35 Hz) frequency ranges in the *x* axis (log scale). Red and blue lines represent the averaged power spectrum in the phasic and tonic patients, respectively. The shaded area represents the area of SE. **(B)** Red and blue bars represent the relative power in GPi and GPe averaged over the delta (a), theta (b), alpha (c), and beta (d) frequency ranges in the phasic and tonic patients. The asterisks indicate a significant difference in GPi and GPe relative power (*p < 0.05 and **p < 0.01). Error bars represent SEs.

Then, the averaged GPi/GPe powers were plotted across each clinical group of the phasic and tonic patients (Figure 2A). As expected, the spectral peaks in the GPi and GPe over the alpha frequency range were observed in the phasic patients. The averaged power over the delta, theta, alpha, and beta frequency ranges in each clinical group showed that the alpha power was significantly higher in the phasic than in the tonic patients in the GPi (*p* < 0.01) (Figure 2Bc). Moreover, within the phasic patients, the alpha power was significantly higher in the GPi than in the GPe (*p* < 0.01, Figure 2Bc). On the other hand, the delta power in the GPi was significantly greater in tonic than in phasic patients (*p* < 0.05, Figure 2Ba). As for the power over the theta and beta frequency ranges, there were no significant differences between the phasic and tonic patients. In addition, within the phasic patients, there were no differences in GPi/GPe oscillatory power over each frequency range between the focal and generalized dystonic patients (Figure S3 in Supplementary Material). These results suggest that delta and alpha resting-state GPi oscillatory activities are differentially characterized by their dominant symptoms (tonic and phasic symptoms).

### Functional Coupling Between Internal and External Segments of the Globus Pallidus

**Figure 3:**
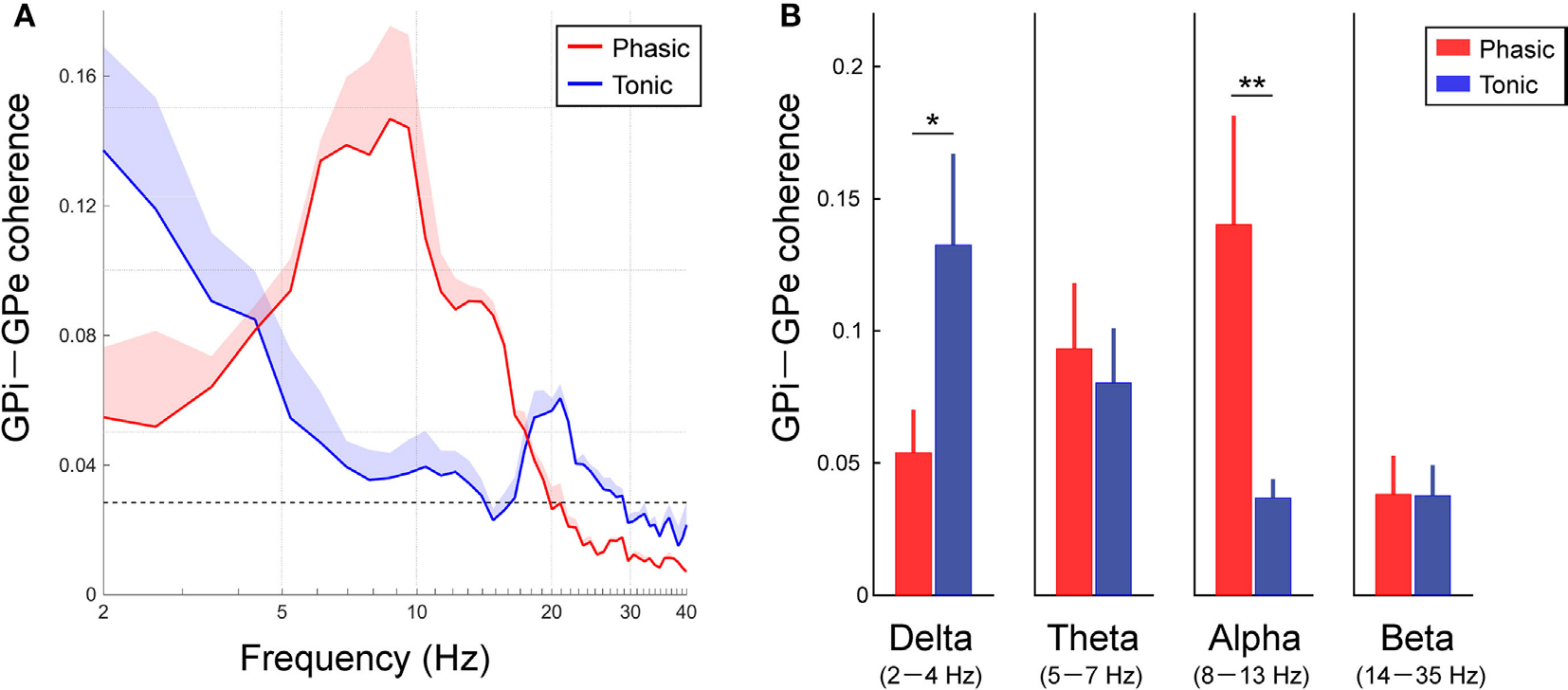
Resting-state GPi–GPe functional couplings in phasic and tonic dystonia patients. **(A)** Red and blue lines represent the averaged GPi–GPe coherence profiles in the phasic and tonic patients, respectively. The *x*-axis shows the log scale. The shaded area represents the SE. The black horizontal dotted line shows the significant level of coherence. Significant coherence is found at the peak around the alpha band in the phasic and tonic patients. **(B)** The mean value of GPi–GPe coherence over the delta (2–4 Hz) (a), theta (5–7 Hz) (b), alpha (8–13 Hz) (c), and beta (14–35 Hz) (d) bands in the phasic (red) and tonic (blue) patients. Error bars represent the SEs. Significant differences in coherence between phasic and tonic symptoms of dystonia are found in the delta and alpha bands (*p < 0.05 and **p < 0.01).

To identify the characteristics of the frequency-specific functional couplings between the GPi and GPe, the averaged coherence between the GPi and GPe was calculated in each clinical group of phasic and tonic patients (Figure 3A). In both groups of dystonic patients, the resting-state GPi–GPe coherence was significant over the broad frequency ranges, including the delta, theta, alpha, and beta bands. In particular, the spectral peaks around the alpha bands emerged in the phasic patients. In the tonic patients, however, there was no alpha peak in GPi–GPe coherence. Instead, the coherence around 20 Hz and lower frequencies including the delta band was prominent (Figure 3A). The averaged coherence values in each frequency band (Figure 3B) suggest that the alpha GPi–GPe coherence was significantly greater in phasic than in tonic patients (*p* < 0.05). Conversely, delta coherence was significantly greater in tonic than in phasic patients (*p* < 0.05). There were no differences in GPi–GPe coherence over the theta and beta bands (Figure 3B). These results suggest that the delta and alpha resting-state functional couplings between GPi and GPe were the characteristic prominent features in the predominant tonic and phasic groups, respectively.

### Functional Coupling Between the Globus Pallidus and Motor Cortex

**Figure 4:**
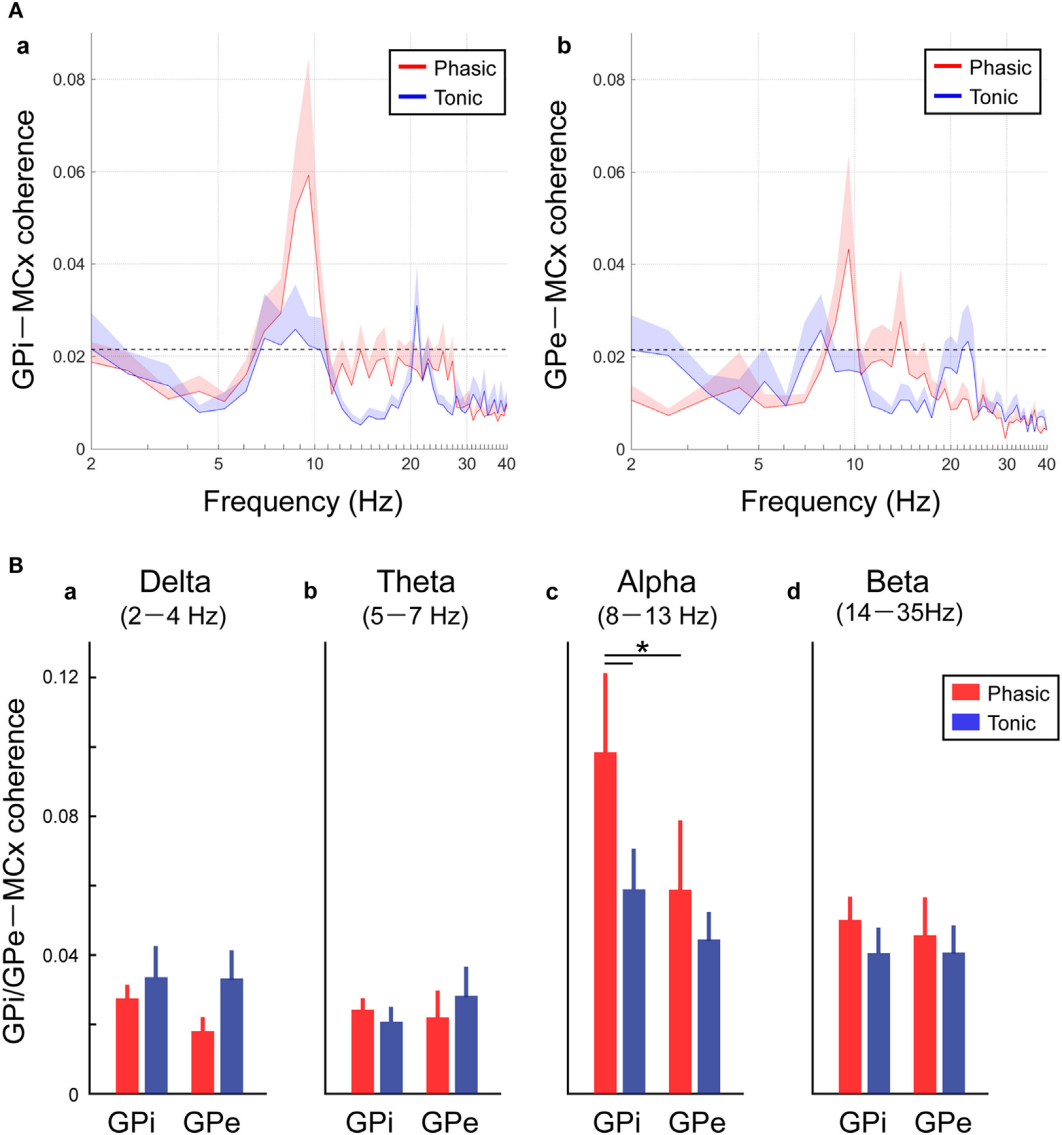
Resting-state GPi-MCX and GPe-MCx coherences in the phasic and tonic patients. **(A)** Red and blue lines represent the averaged GPi-MCx coherence (a) and GPe-MCx coherence (b) in phasic and tonic patients, respectively. The shaded areas represent the SE. The black horizontal dotted lines show the significant level of coherence. The peaks of the significant GPi-MCx and GPe-MCx coherences are found in the alpha range, especially in phasic patients. **(B)** Red and blue bars represent the averaged value of GPi-MCx and GPe-MCx coherences over the delta (2–4 Hz) (a), theta (5–7 Hz) (b), alpha (8–13 Hz) (c), and beta (14–35 Hz) (d) bands in phasic and tonic patients, respectively. The alpha GPi–MCx coherences are higher in phasic than in tonic patients (*p < 0.05).

As well as the functional couplings locally within the GPi and GPe, the coherences between the GP and MCx (i.e., GPi–MCx and GPe–MCx coherence) at rest were also investigated and compared between the phasic and tonic groups (Figure 4). The alpha spectral peaks of GPi/GPe–MCx coherences were significant over the alpha frequency range in phasic patients, but they were not prominent in tonic patients (Figure 4A). The averaged GPi/GPe–MCx coherences in each frequency range are shown in Figure 4B, suggesting that the alpha GPi–MCx coherences were significantly higher in phasic than in tonic patients (*p* < 0.05, Figure 4Bc). Within the phasic patients, the alpha GPi–MCx coherence was also significantly higher than the GPe–MCx coherence (*p* < 0.05, Figure 4Bc). As for the other frequency ranges (delta, theta, and beta), there were no significant changes in GPi–MCx and GPe–MCx coherences between the phasic and tonic patients (Figure 4Ba,b,d). Moreover, significant positive correlations were found between the alpha GPi power and the alpha GPi coherence with the MCx, and between the alpha GPe power and the alpha GPe coherence with the MCx across the phasic patients (*p* < 0.05, shown in Figure S4 in Supplementary Material). However, there were no significant correlations over the other frequency ranges (delta, theta, and beta), suggesting that the resting-state alpha GPi/GPe oscillatory activities are related to GP–MCx functional coupling, and such alpha GP–MCx coherence is a distinctive feature in phasic patients.

## Discussion

**Figure 5:**
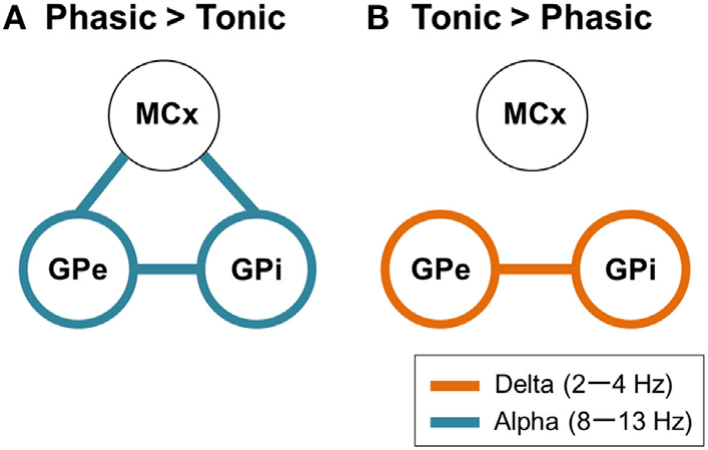
Schematic design of frequency-specific characteristic GPi and GPe oscillatory activities and functional couplings segregated by dystonic symptoms. **(A)** The schematic power and coherence profiles that are more prominent in phasic than in tonic patients. In summary, the alpha GPi power (blue circles) and the alpha GPi coherences with the GPe and MCx (blue lines) are the prominent features in phasic patients. **(B)** The schematic power and coherence profiles that are more prominent in tonic than in phasic patients. In summary, the delta GPi power (orange circles) and the delta GPi coherences with GPe (orange line) are the prominent features in tonic patients.

In the present study, it was shown that the resting-state GPi/GPe oscillatory activities and the functional couplings across GPi, GPe, and MCx represent the frequency-specific characteristics that depend on whether the dystonic patients’ symptoms are predominant phasic or tonic. In phasic patients, prominent peaks in the GPi oscillatory activities were observed in the alpha frequency range, and this oscillation was functionally coupled across the GPi, GPe, and MCx (Figures 3, 4, and 5A). On the other hand, in tonic patients, the delta oscillatory activities in the GPi emerged, and delta GPi–GPe functional coupling was greater in tonic than in phasic patients (Figures 3 and 5B). These results suggest that the symptom-specific delta/alpha pallidal oscillatory activities and the functional couplings among the GPi, GPe, and MCx are present at rest, supporting the notion that different pathophysiological mechanisms may be underlying the predominant phasic and tonic symptoms.

So far, the previous research with postoperative LFP recordings has suggested that the GP oscillatory activities in the low-frequency component (4–13 Hz) including the theta (4–7 Hz) and alpha (8–13 Hz) ranges were critically related to primary dystonia (18, 30) and myoclonic dystonia (31). Liu et al. (4) further found that the distinct features of the subtypes of phasic and tonic symptoms were characterized by GPi-muscular coherence at this low frequency. They found that, during involuntary dystonic movements, low-frequency GPi–muscular coherence emerged in phasic patients, but not in tonic dystonic patients. Notably, the present study showed that distinct features of predominant phasic and tonic symptoms could be extracted from the pallidal oscillations in the resting-state without any involuntary or voluntary movements. That is, the GPi power and coherence with the GPe and MCx over the two different frequency ranges (delta and alpha bands) were critical for segregating the predominant tonic and phasic symptoms, respectively (Figure 5).

### Alpha GPi Oscillations and Coherence With the MCx in Phasic Symptoms of Dystonia

Many studies have reported that when phasic and rhythmic dystonic movements were present, significant coherence between the GPi and affected muscle was seen over the bursting frequency or the first harmonic frequency including the alpha frequency bands (4, 32). These alpha GPi–muscle coherences were suppressed after high-frequency pallidal stimulation in patients with phasic symptoms (5), suggesting a causal link between the alpha pallidal oscillations and the pathophysiology of phasic symptoms. In line with the previous studies, the alpha GPi oscillation was prominent in the predominant phasic symptom patients, and the alpha GPi oscillation was functionally coupled with the GPe, as well as the MCx, in the present study. The previous studies in DYT1 dystonia model mice (33) and a human cervical dystonia patient (34, 35) showed activity changes in the GPi and GPe, such as reduced spontaneous firing rates with burst and long-lasting inhibition evoked by electrical stimulation to the MCx. This change may indicate the increased activity in the GPi through the cortico–striato–GPi direct and cortico–striato–GPe indirect pathways (36). In this respect, the alpha resting-state GPi–GPe–MCx functional couplings may represent the abnormal cortico-BG circuits probably *via* the direct and indirect neural pathways, which had a central role for patients with phasic symptoms.

### Delta GPi Oscillations and Coherence With the GPe in Tonic Symptom of Dystonia

On the other hand, the present study also demonstrated that delta GPi oscillatory activities (Figure 2Ba) and delta GPi–GPe coherence (Figure 3B) were the prominent features in tonic patients. To the best of our knowledge, few studies have examined the characteristics of the GPi/GPe focused on predominant tonic symptom patients. Liu et al. (4) reported the absence of alpha coherence between the MCx and the affected hypertonic dystonic muscles. In line with the previous study, the present study demonstrated lack of coherence between the GPi/GPe and MCx in tonic patients. On the other, some dystonia improved by stereotactic surgery of the ventral part of the thalamus, which anatomically projects to the MCx. Horisawa et al. (37) reported the outcome of the ventro-oral thalamotomy in 15 patients with musician’s dystonia. Hand dystonia improved and the effects are sustained over a long period of time. The 74% of the patients included in this study had generalized symptoms with truncal dystonia. The pathophysiological state of tonic symptom in dystonia should be not unique. It is supposed that the neural circuit on tonic symptoms of dystonia vary depending on the body part and pathophysiology. The neural circuit of tonic symptoms of dystonia vary depending on the phenotype of the body part or form of dystonia. The neural circuits involved in the hand task-specific dystonia are thalamo–cortical pathway. But, the result in the study implies that the other neural pathways that are not mediated *via* the MCx may cause the tonic symptoms of dystonia. Recent study suggested that the cerebellar dysfunction induced involuntary movement like dystonia. The inositol 1,4,5-trisphosphate receptors (IP3Rs) located in the cerebellum and brainstem are important for diverse physiological phenomena including gene expression, development, growth, and neural plastic-secretion. The knockout mice lacking type 1 IP3Rs showed dystonic-like behavior, and the Purkinje cell firing patterns were coupled to hypertonic dystonic movements (38). Abnormal connectivity between the GPi and brainstem (39, 40) or the cerebellum (40) was also reported in patients with primary dystonia. In addition, it was shown that brainstem lesions cause dystonic symptoms (41). Anatomical and imaging studies further suggested that these cerebellum–BG–brainstem neuronal pathways are mediated by the pedunculopontine tegmental nucleus [PPN (42–44)], which has been known to contribute to automatic control of movements, such as adjustment of postural muscle tone during locomotion (45–47). Although such a hypothesis still lacks proof, the results imply that the abnormal cerebellar outputs directly sent to the spinal cord *via* the BG, red nucleus, or reticular formation, which were not directly mediated *via* the MCx, might have a central role in hypertonic dystonic symptoms.

As for the delta GPi oscillations, few studies have demonstrated them in patients with dystonia. Originally, low-frequency delta oscillations were confirmed in slow-wave sleep (48, 49). Recently, it has been suggested that the delta cortical oscillatory dynamics are common across the states, not only during sedation and sleep but also during movement kinematics (50). In primary dystonia, the delta GPi power is suggested to increase following both active and passive movements (51). Clinically, it is known that dystonic symptoms become more evident during passive or active movements in tonic patients, such as with abnormal posture, and it would be plausible that resting-state GPi delta power is related to predominant tonic symptoms. Further studies will need to investigate the delta-specific changes in pallidal oscillatory activities and their functional couplings during abnormal movements to elucidate the pathophysiology of the clinical phenotypes of dystonia.

Despite the lack of dystonic symptoms, abnormal oscillations related to the tonic and phasic symptoms were observed. Therefore, the abnormal oscillations indicate the pathophysiological abnormalities of dystonia in the central nervous system.

### Limitations of the Study

Finally, the present study has important limitations. First, in the experimental designs, age was not matched between the two groups (*p* < 0.05). In addition, BFMDRS scores were higher in the tonic group than in the phasic group (*p* < 0.01). With these significant differences between the two groups, the results obtained from the dystonic patients should be interpreted carefully. Second, for a group comparison of each clinical phenotype, a sufficient number of patients is needed. Thus, the present results with only 10 and 9 patients in each subtype of dystonia may still be considered preliminary. However, to the best of our knowledge, the present study was the largest group study to investigate simultaneous recordings of pallidal LFPs and MCx EEGs in relation to distinct subtypes of dystonia.

Overall, the present study demonstrated that frequency-specific pallidal oscillatory activities and their functional couplings distinguished the phenotypes of dystonia that are present at rest, thus, providing further support for the hypothesis that different cortico-pallidal pathophysiological mechanisms may be underlying the predominant phasic and tonic symptoms of dystonia.

## Ethics Statement

The present study was approved by the ethics committees of the Tokyo Metropolitan Neurological Hospital (Tokyo, Japan) and the Faculty of Science and Technology, Keio University (Kanagawa, Japan). The study and publication were explained to all patients and family, and the consent obtained from the patients was both informed and written.

## Author Contributions

FY: data collection (DC), data analysis (DA), operation (OP), manuscript writing (MW), and management of patients (MP). KKato: DC, DA, and MW. HI: OP. TK: MP, DC, and OP. KKimura: OP and MP. AY: DC, OP, and MP. RO: OP and MP. MT: OP. SK: MP. JU: DA and MW.

## Conflict of Interest Statement

The authors declare that the research was conducted in the absence of any commercial or financial relationships that could be construed as a potential conflict of interest.

## Abbreviations

DBS, deep brain stimulation; GPi, globus pallidus internus; GPe, globus pallidus externus; LFP, local field potential; MCx, motor cortex; EEG, electroencephalogram; BFMDRS, Burke-Fahn-Marsden Dystonia Rating Scale; Tonic patient, patient with predominant tonic symptoms; Phasic patient, patients with predominant phasic symptoms; BG, basal ganglia; PD, Parkinson’s disease; MLE, micro lesion effect.

## References

[1] Albanese A Bhatia K Bressman SB Delong MR Fahn S Fung VS Phenomenology and classification of dystonia: a consensus update. Mov Disord (2013) 28(7):863–73. 10.1002/mds.25475 23649720 PMC3729880

[2] Berardelli A Rothwell JC Hallett M Thompson PD Manfredi M Marsden CD. The pathophysiology of primary dystonia. Brain (1998) 121:1195–212. 10.1093/brain/121.7.1195 9679773

[3] Vercueil L Pollak P Fraix V Caputo E Moro E Benazzouz A Deep brain stimulation in the treatment of severe dystonia. J Neurol (2001) 248(8):695–700. 10.1007/s004150170116 11569899

[4] Liu X Yianni J Wang S Bain PG Stein JF Aziz TZ. Different mechanisms may generate sustained hypertonic and rhythmic bursting muscle activity in idiopathic dystonia. Exp Neurol (2006) 198(1):204–13. 10.1016/j.expneurol.2005.11.018 16410002

[5] Barow E Neumann WJ Brücke C Huebl J Horn A Brown P Deep brain stimulation suppresses pallidal low frequency activity in patients with phasic dystonic movements. Brain (2014) 137:3012–24. 10.1093/brain/awu258 25212852 PMC4813762

[6] Coubes P Roubertie A Vayssiere N Hemm S Echenne B. Treatment of DYT1-generalised dystonia by stimulation of the internal globus pallidus. Lancet (2000) 355:2220–1. 10.1016/S0140-6736(00)02410-7 10881900

[7] Coubes P Cif L El Fertit H Hemm S Vayssiere N Serrat S Electrical stimulation of the globus pallidus internus in patients with primary generalized dystonia: long-term results. J Neurosurg (2004) 101(2):189–94. 10.3171/jns.2004.101.2.0189 15309907

[8] Vidailhet M Vercueil L Houeto JL Krystkowiak P Benabid AL Cornu P Bilateral deep-brain stimulation of the globus pallidus in primary generalized dystonia. N Engl J Med (2005) 352:459–67. 10.1056/NEJMoa042187 15689584

[9] Vidailhet M Vercueil L Houeto JL Krystkowiak P Lagrange C Yelnik J Bilateral, pallidal, deep-brain stimulation in primary generalised dystonia: a prospective 3 year follow-up study. Lancet Neurol (2007) 6(3):223–9. 10.1016/S1474-4422(07)70035-2 17303528

[10] Kupsch A Benecke R Muller J Trottenberg T Schneider GH Poewe W Pallidal deep-brain stimulation in primary generalized or segmental dystonia. N Engl J Med (2006) 355:1978–90. 10.1056/NEJMoa063618 17093249

[11] Ostrem JL Starr PA. Treatment of dystonia with deep brain stimulation. Neurotherapeutics (2008) 5:320–30. 10.1016/j.nurt.2008.01.002 18394573 PMC5084173

[12] Volkmann J Wolters A Kupsch A Muller J Kuhn AA Schneider GH Pallidal deep brain stimulation in patients with primary generalized or segmental dystonia: 5-year follow-up of a randomised trial. Lancet Neurol (2012) 11:1029–38. 10.1016/S1474-4422(12)70257-0 23123071

[13] Yianni J Bain PG Gregory RP Nandi D Joint C Scott RB Post-operative progress of dystonia patients following globus pallidus internus deep brain stimulation. Eur J Neurol (2003) 10:239–47. 10.1046/j.1468-1331.2003.00592.x 12752397

[14] Krauss JK Yianni J Loher TJ Aziz TZ. Deep brain stimulation for dystonia. J Clin Neurophysiol (2004) 21(1):18–30. 10.1097/00004691-200401000-00004 15097291

[15] Hallett M. Neurophysiology of dystonia: the role of inhibition. Neurobiol Dis (2011) 42(2):177–84. 10.1016/j.nbd.2010.08.025 20817092 PMC3016461

[16] Liu X Griffin IC Parkin SG Miall RC Rowe JG Gregory RP Involvement of the medial pallidum in focal myoclonic dystonia: a clinical and neurophysiological case study. Mov Disord (2002) 17:346–53. 10.1002/mds.10038 11921122

[17] Silberstein P Kuhn AA Kupsch A Trottenberg T Krauss JK Wohrle JC Patterning of globus pallidus local field potentials differs between Parkinson’s disease and dystonia. Brain (2003) 126:2597–608. 10.1093/brain/awg267 12937079

[18] Chen CC Kühn AA Hoffmann KT Kupsch A Schneider GH Trottenberg T Oscillatory pallidal local field potential activity correlates with involuntary EMG in dystonia. Neurology (2006) 66:418–20. 10.1212/01.wnl.0000196470.00165.7d 16476944

[19] Brown P. Oscillatory nature of human basal ganglia activity: relationship to the pathophysiology of Parkinson’s disease. Mov Disord (2003) 18(4):357–63. 10.1002/mds.10358 12671940

[20] Kato K Yokochi F Taniguchi M Okiyama R Kawasaki T Kimura K Bilateral coherence between motor cortices and subthalamic nuclei in patients with Parkinson’s disease. Clin Neurophysiol (2015) 126(10):1941–50. 10.1016/j.clinph.2014.12.007 25591829

[21] Oswal A Beudel M Zrinzo L Limousin P Hariz M Foltynie T Deep brain stimulation modulates synchrony within spatially and spectrally distinct resting state networks in Parkinson’s disease. Brain (2016) 139:1482–96. 10.1093/brain/aww048 27017189 PMC4845255

[22] Hinkley LB Sekihara K Owen JP Westlake KP Byl NN Nagarajan SS. Complex-value coherence mapping reveals novel abnormal resting-state functional connectivity networks in task-specific focal hand dystonia. Front Neurol (2013) 4:149. 10.3389/fneur.2013.00149 24133480 PMC3794296

[23] Peller M Zeuner KE Munchau A Quartarone A Weiss M Knutzen A The basal ganglia are hyperactive during the discrimination of tactile stimuli in writer’s cramp. Brain (2006) 129:2697–708. 10.1093/brain/awl181 16854945

[24] Halliday DM Rosenberg JR Amjad AM Breeze P Conway BA Farmer SF A framework for the analysis of mixed time series/point process data-theory and application to the study of physiological tremor, single motor unit discharges and electromyograms. Prog Biophys Mol Biol (1995) 64:237–78. 10.1016/S0079-6107(96)00009-0 8987386

[25] de Solages C Hill BC Koop MM Henderson JM Bronte-Stewart H Bilateral symmetry and coherence of subthalamic nuclei beta band activity in Parkinson’s disease. Exp Neurol (2010) 221(1):260–6. 10.1016/j.expneurol.2009.11.012 19944098

[26] Höller Y Butz K Thomschewski A Schmid E Uhl A Bathke AC Reliability of EEG interactions differs between measures and is specific for neurological diseases. Front Hum Neurosci (2017) 11:350. 10.3389/fnhum.2017.00350 28725190 PMC5496950

[27] Kato K Yokochi F Iwamuro H Kawasaki T Hamada K Isoo A Frequency-specific synchronization in the bilateral subthalamic nuclei depending on voluntary muscle contraction and relaxation in patients with Parkinson’s disease. Front Hum Neurosci (2016) 10:131. 10.3389/fnhum.2016.00131 27064969 PMC4811912

[28] Rosenberg JR Amjad AM Breeze P Brillinger DR Halliday DM The Fourier approach to the identification of functional coupling between neuronal spike trains. Prog Biophys Mol Biol (1989) 53:1–31. 10.1016/0079-6107(89)90004-7 2682781

[29] Cersosimo MG Raina GB Benarroch EE Piedimonte F Alemán GG Micheli FE. Micro lesion effect of the globus pallidus internus and outcome with deep brain stimulation in patients with Parkinson disease and dystonia. Mov Disord (2009) 24(10):1488–93. 10.1002/mds.22641 19475579

[30] Chen CC Kühn AA Trottenberg T Kupsch A Schneider GH Brown P Neuronal activity in globus pallidus interna can be synchronized to local field potential activity over 3–12 Hz in patients with dystonia. Exp Neurol (2006) 202(2):480–6. 10.1016/j.expneurol.2006.07.011 16930593

[31] Foncke EM Bour LJ Speelman JD Koelman JH Tijssen MA. Local field potentials and oscillatory activity of the internal globus pallidus in myoclonus-dystonia. Mov Disord (2007) 22(3):369–76. 10.1002/mds.21284 17216649

[32] Sharott A Grosse P Kühn AA Salih F Engel AK Kupsch A Is the synchronization between pallidal and muscle activity in primary dystonia due to peripheral afferance or a motor drive? Brain (2008) 131:473–84. 10.1093/brain/awm324 18178569

[33] Chiken S Shashidharan P Nambu A. Cortically evoked long-lasting inhibition of pallidal neurons in a transgenic mouse model of dystonia. J Neurosci (2008) 28(51):13967–77. 10.1523/JNEUROSCI.3834-08.2008 19091985 PMC2702121

[34] Moll CK Galindo-Leon E Sharott A Gulberti A Buhmann C Koeppen JA Asymmetric pallidal neuronal activity in patients with cervical dystonia. Front Syst Neurosci (2014) 8:15. 10.3389/fnsys.2014.00015 24574981 PMC3920073

[35] Nishibayashi H Ogura M Kakishita K Tanaka S Tachibana Y Nambu A Cortically evoked responses of human pallidal neurons recorded during stereotactic neurosurgery. Mov Disord (2011) 26:469–76. 10.1002/mds.23502 21312279

[36] Nambu A Chiken S Shashidharan P Nishibayashi H Ogura M Kakishita K Reduced pallidal output causes dystonia. Front Syst Neurosci (2011) 5:89. 10.3389/fnsys.2011.00089 22164134 PMC3224972

[37] Horisawa S Taira T Goto S Ochiai T Nakajima T. Long-term improvement of musician’s dystonia after stereotactic ventro-oral thalamotomy. Ann Neurol (2013) 74(5):627–9. 10.1002/ana.23877 23463596

[38] Hisatsune C Miyamoto H Hirono M Yamaguchi N Sugawara T Ogawa N IP3R1 deficiency in the cerebellum/brainstem causes basal ganglia-independent dystonia by triggering tonic Purkinje cell firings in mice. Front Neural Circuits (2013) 7:156. 10.3389/fncir.2013.00156 24109434 PMC3790101

[39] Blood AJ Kuster JK Woodman SC Kirlic N Makhlouf ML Multhaupt-Buell TJ Evidence for altered basal ganglia-brainstem connections in cervical dystonia. PLoS One (2012) 7:e31654. 10.1371/journal.pone.0031654 22384048 PMC3285161

[40] Neumann WJ Jha A Bock A Huebl J Horn A Schneider GH Cortico-pallidal oscillatory connectivity in patients with dystonia. Brain (2015) 138:1894–906. 10.1093/brain/awv109 25935723

[41] Vidailhet M Dupel C Lehericy S Remy P Dormont D Serdaru M Dopaminergic dysfunction in midbrain dystonia: anatomoclinical study using 3-dimensional magnetic resonance imaging and fluorodopa F 18 positron emission tomography. Arch Neurol (1999) 56:982–9. 10.1001/archneur.56.8.982 10448804

[42] Vitek JL. Pathophysiology of dystonia: a neuronal model. Mov Disord (2002) 17(Suppl 3):S49–62. 10.1002/mds.10142 11948755

[43] Niethammer M Carbon M Argyelan M Eidelberg D. Hereditary dystonia as a neurodevelopmental circuit disorder: evidence from neuroimaging. Neurobiol Dis (2011) 42(2):202–9. 10.1016/j.nbd.2010.10.010 20965251 PMC3062649

[44] Zhang J Wang ZI Baker KB Vitek JL. Effect of globus pallidus internus stimulation on neuronal activity in the pedunculopontine tegmental nucleus in the primate model of Parkinson’s disease. Exp Neurol (2012) 233(1):575–80; *Ann Neurol* (2013) 74(5):648–54. 10.1016/j.expneurol.2011.07.007 21821025 PMC3536473

[45] Lee MS Rinne JO Marsden CD. The pedunculopontine nucleus: its role in the genesis of movement disorders. Yonsei Med J (2000) 41(2):167–84. 10.3349/ymj.2000.41.2.167 10817016

[46] Takakusaki K Saitoh K Harada H Kashiwayanagi M Role of basal ganglia-brainstem pathways in the control of motor behaviors. Neurosci Res (2004) 50(2):137–51. 10.1016/j.neures.2004.06.015 15380321

[47] Takakusaki K Habaguchi T Saitoh K Kohyama J Changes in the excitability of hindlimb motoneurons during muscular atonia induced by stimulating the pedunculopontine tegmental nucleus in cats. Neuroscience (2004) 124(2):467–80. 10.1016/j.neuroscience.2003.12.016 14980396

[48] Colrain IM. The K-complex: a 7-decade history. Sleep (2005) 28(2):255–73. 10.1093/sleep/28.2.255 16171251

[49] Cash SS Halgren E Dehghani N Rossetti AO Thesen T Wang C The human K-complex represents an isolated cortical down-state. Science (2009) 324(5930):1084–7. 10.1126/science.1169626 19461004 PMC3715654

[50] Hall TM de Carvalho F Jackson A. A common structure underlies low-frequency cortical dynamics in movement, sleep, and sedation. Neuron (2014) 83(5):1185–99. 10.1016/j.neuron.2014.07.022 25132467 PMC4157580

[51] Liu X Wang S Yianni J Nandi D Bain PG Gregory R The sensory and motor representation of synchronized oscillations in the globus pallidus in patients with primary dystonia. Brain (2008) 131:1562–73. 10.1093/brain/awn083 18487278

