# Supplementary material for: Resting-State Pallidal-Cortical Oscillatory Couplings in Patients With Predominant Phasic and Tonic Dystonia
Source: Front Neurol. 2018 May 31;9:375. doi: 10.3389/fneur.2018.00375 (PMC5990626; doi:10.3389/fneur.2018.00375)
Supplement: Supplementary file 1 [file presentation_1.PDF]

Supplementary materials

Supplementary Fig. 1

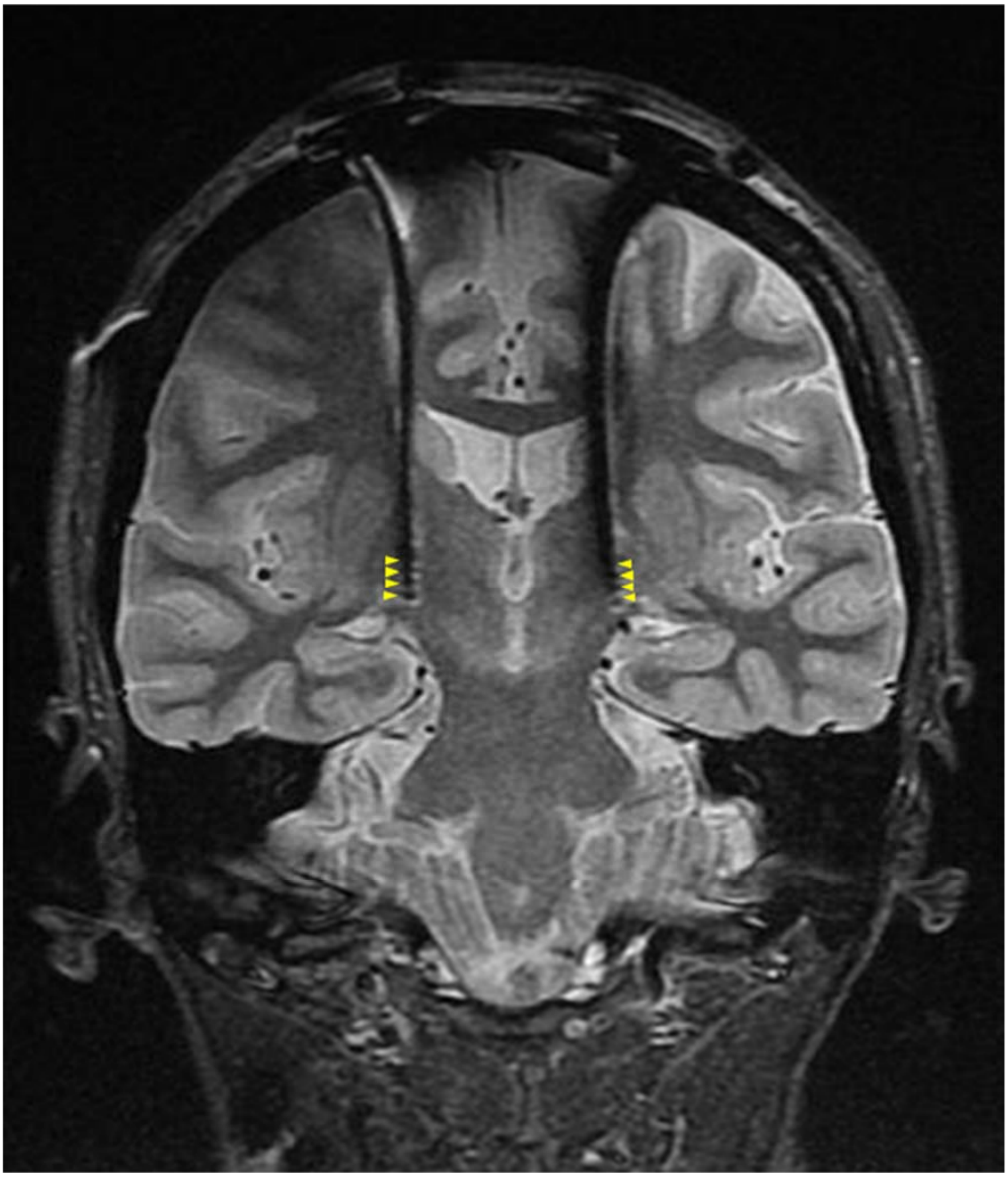

Supplementary Fig 1. MRI of DYT11 patient (Patient 1) underwent bilateral GPi DBS. The yellow triangles represent the positions of contacts of implanted DBS electrodes, ensuring that the DBS electrodes target the GPi bilaterally.

Supplementary Fig. 2

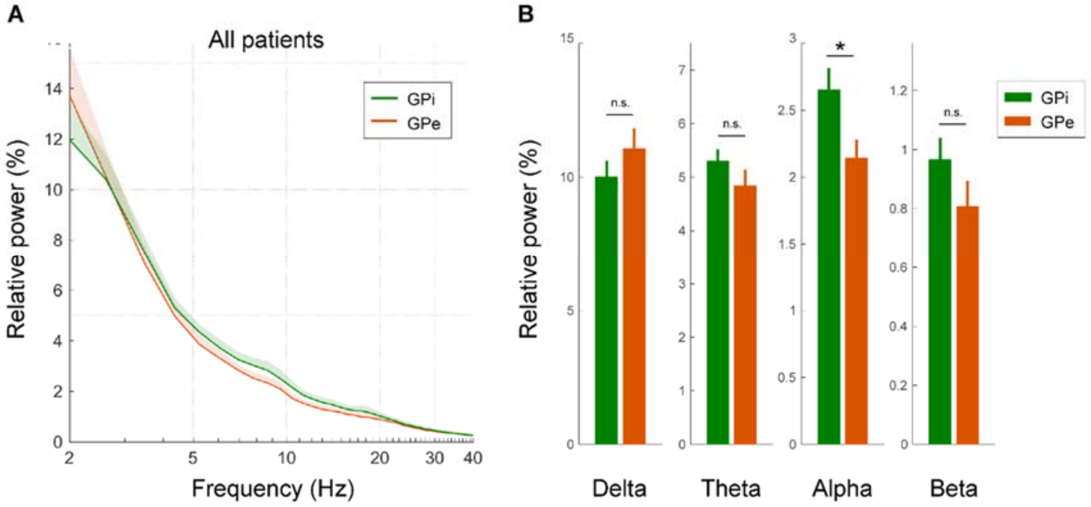

Supplementary Fig 2. Distinct resting-state GPi and GPe oscillatory power spectral distributions in patients with the primary dystonia. A: The averaged relative power in GPi (green) and GPe (orange) over the delta, theta, alpha, and beta frequency ranges in all the dystonic patients are represented in the x axis of a log scale. The shaded area represents the area of standard error. **B**: Each bar represents the relative power in GPi (green) and GPe (orange) averaged over the delta, theta, alpha, and beta frequency ranges across all the dystonic patients. The asterisks indicate a significant difference in GPi and GPe relative power (∗, $p < 0.05$). Error bars represent standard errors.

Supplementary Fig. 3

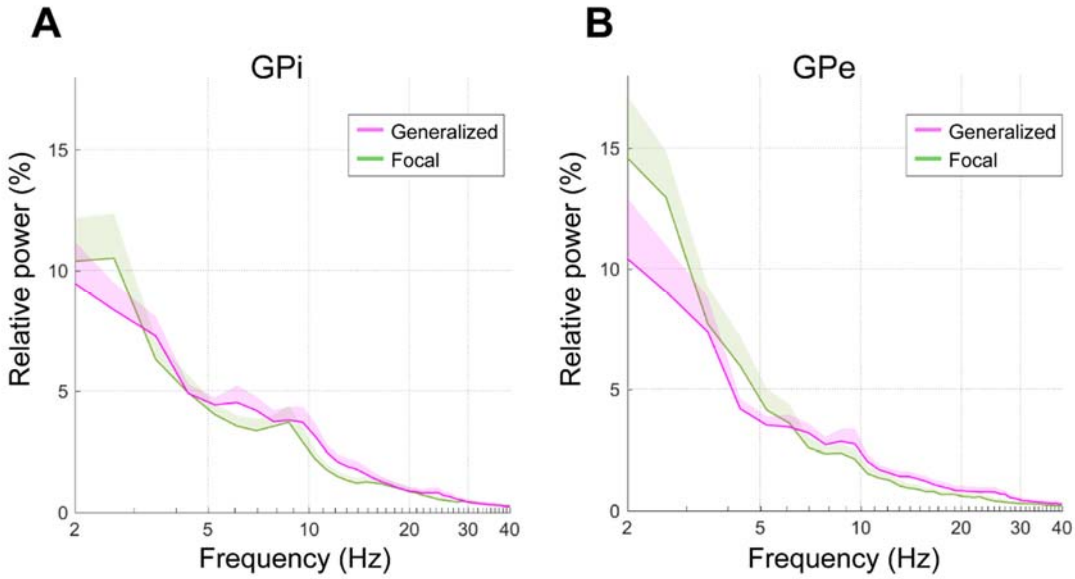

Supplementary Fig 3. Resting-state GPi and GPe oscillatory power spectral distributions in patients with the generalized and focal dystonia. A: The pink and green lines represent the averaged relative power in GPi (**A**) and GPe (**B**) over the delta, theta, alpha, and beta frequency ranges in patients with generalized and focal dystonia, respectively. The x axis shows a log scale. The shaded area represents the area of standard error. There is no mean spectral changes between generalized and focal dystonia over the delta, theta, alpha, and beta bands.

Supplementary Fig. 4

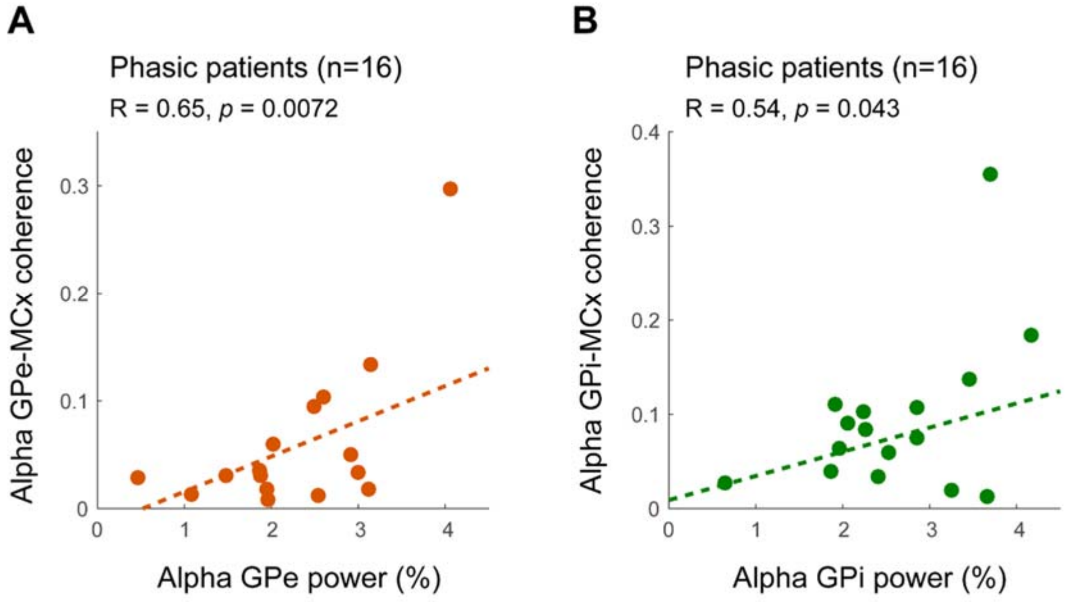

Supplementary Fig 4. Positive correlation between alpha GPi/GPe power and GPi/GPe－MCx coherence in phasic patients. In patients with the predominant phasic symptoms, there are significant positive correlations between the alpha GPi power and GPi－MCx coherence (**A**) and between the alpha GPe power and GPe－MCx coherence (**B**).
